# Trends in Coronavirus Disease 2019 Mortality Within a US Academic Health System, 2020–2025

**DOI:** 10.1093/ofid/ofag138

**Published:** 2026-03-14

**Authors:** Nicholas A Turner, Jason E Stout, Jeffrey D Jenks

**Affiliations:** Department of Medicine, Division of Infectious Diseases, Duke University School of Medicine, Durham, North Carolina, USA; Department of Medicine, Division of Infectious Diseases, Duke University School of Medicine, Durham, North Carolina, USA; Department of Medicine, Division of Infectious Diseases, Duke University School of Medicine, Durham, North Carolina, USA; Durham County Department of Public Health, Durham, North Carolina, USA

**Keywords:** coronavirus disease 2019 (COVID-19)

## Abstract

Coronavirus disease 2019 (COVID-19) was initially associated with higher mortality than other respiratory viruses. We assessed trends in mortality following a positive COVID-19 test within a single academic health system. Among individuals with COVID-19, 30-day crude and adjusted mortality rates have decreased but remain numerically similar to higher than influenza.

Coronavirus disease 2019 (COVID-19) was associated with a high initial mortality rate, rapidly becoming the top age-standardized cause of death in 2021. By 2023, COVID-19 fell from the top 20 causes of mortality [1]. Studies have identified multiple contributors to reduced COVID-19 mortality over time—including the rise of less virulent variants (the Omicron variant was temporally associated with a sharp reduction in mortality), development of highly effective vaccines (associated with a >60% reduction in mortality even after the rise of Omicron), immunity due to past infection (seroprevalence surveys from blood banks show high rates of combined infection- and vaccine-induced antibodies), and improvements in clinical care [1–4].

As infection prevention decisions at both individual and population levels are informed partly by an up-to-date understanding of risk, we sought to reassess COVID-19 mortality rates using a 5-year (2020–2025) testing cohort from a large academic medical center. To be both timely and large scale, it was designed as a pragmatic longitudinal survey of data amenable to electronic query. Influenza A was used as a comparator, as this tends to be a familiar “reference infection” for many patients, clinicians, and policymakers. First, we assessed for changes in 30-day mortality following a positive COVID-19 test over time—while adjusting for potential confounders among individuals tested. Second, we compared 30-day mortality rates following a positive COVID-19 test against a positive influenza A test—again with adjustment for potential confounders among individuals tested.

## METHODS

### Data Collection

Cases were identified by electronically querying for a positive severe acute respiratory syndrome coronavirus 2 or influenza A test among adults (age ≥18 years) processed by the Duke Clinical Microbiology Laboratory (which serves 1 large academic health system) between 1 January 2020 and 31 August 2025. The query was conducted on 2 October 2025 to ensure a 30-day follow-up beyond the last test. Tests with a listed indication of “administrative” (eg, screening prior to nursing home transfer), preprocedure screening, or asymptomatic screening were excluded. Any repeat positives within 28 days of a prior test were considered duplicates and excluded. Individuals who tested positive for both COVID-19 and influenza A were excluded to avoid ambiguity in attributing risk association. History of hematopoietic or solid organ transplant and components of the Charlson comorbidity index were queried electronically using the Duke Enterprise Data Unified Content Explorer tool [5, 6].

Study design was reviewed by Duke University Health System's institutional review board and determined to be exempt, with waiver of consent for data collection.

### Statistical Modeling

To adjust for individual-level confounders as COVID-19 testing practices evolved over time, we constructed multivariable logistic regression models for 30-day mortality adjusting for age, sex, transplant status, and individual components of the Charlson comorbidity index. The Charlson index has previously been shown to correlate with COVID-19 mortality [7–9]. Laboratory-based predictors were not included, as the majority of tested subjects did not have laboratory testing available. To account for the fact that individuals might have been tested more than once (violating the independence assumption), we used generalized estimating equations with events clustered by patient and robust standard error estimates [10]. Continuous variables were assessed for linearity by comparing to restricted cubic splines. Collinearity was assessed by the variance inflation factor. Stability of primary effects was assured by assessing an array of models for each outcome (univariate, age-adjusted, and full models).

Trends in COVID-19 mortality (aim 1) were estimated using both univariate (unadjusted) and multivariate (adjusted) odds ratios (ORs) with 2020 serving as the reference year. For aim 2, univariate (unadjusted) and multivariate (adjusted) ORs were calculated for 30-day mortality following either a positive COVID-19 or influenza A test and stratified by year. To assess the potential impact of differing testing practices for COVID-19 and influenza A, we conducted a sensitivity analysis comparing 30-day mortality rates among the subset of patients tested with a basic respiratory viral battery (a single orderable test including influenza A, COVID-19, and respiratory syncytial virus, which became available at our institution in 2024). All models were constructed in R (version 4.4.1, https://cran.r.project.org) using geepack.

## RESULTS

We retrieved 75 018 unique COVID-19 tests and 24 671 unique influenza A tests among individuals age 18 and older between January 2020 and August 2025. Age- and comorbidity-adjusted models for 30-day mortality exhibited strong discriminatory performance for both COVID-19 and influenza A (*C*-statistics 0.89–0.91).

**Figure 1.**
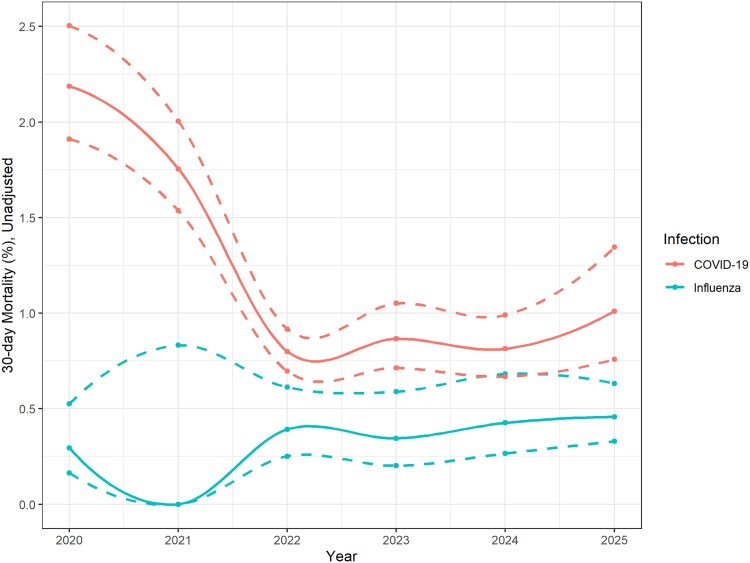
Crude 30-d mortality following a positive test for COVID-19 or influenza A, 2020–2025. Dashed lines represent 95% confidence intervals.

Crude 30-day mortality following a positive COVID-19 test was 2.2% in 2020, then decreased before stabilizing to 0.8%–1.0% from 2023 onward (Figure 1). The population tested for COVID-19 appeared to change over time as well, with generally lower median age and Charlson scores in 2020 compared with later years (Table 1). Both unadjusted and adjusted odds of 30-day mortality following COVID-19 infection decreased since 2020.

**Table 1.** Trends in Crude and Adjusted 30-Day Mortality Following a Positive Test for Influenza A or COVID-19, 2020–2025

Year	Cases	Age, median (IQR)	Male, n (%)	Charlson score >1, n (%)	Crude 30-d mortality, n (%)	Unadjusted OR for 30-d mortality (95% CI)	Adjusted OR for 30-d mortality (95% CI)
*Trends in crude and adjusted 30-d COVID-19 mortality, 2020−2025*							
2020	9371	43 (30–57)	4396 (46.9)	1715 (18.3)	205 (2.2)	**Ref.**	**Ref.**
2021	12 134	42 (29–57)	5272 (43.4)	2513 (20.7)	213 (1.8)	**0.79 (.64–.99)**	**0.76 (.60–.96)**
2022	25 265	46 (32–61)	9676 (38.3)	6050 (23.9)	202 (0.8)	**0.36 (.29–.45)**	**0.24 (.19–.30)**
2023	11 652	53 (37–68)	4257 (36.5)	3526 (30.3)	101 (0.9)	**0.39 (.30–.50)**	**0.19 (.14–.25)**
2024	12 046	53 (37–68)	4374 (36.3)	3789 (31.5)	98 (0.8)	**0.36 (.28–.47)**	**0.16 (.12–.21)**
2025	4550	53 (36–69)	1596 (35.1)	1529 (33.6)	46 (1.0)	**0.45 (.32–.64)**	**0.18 (.13–.27)**
*Comparative 30-d mortality rates following a positive test for influenza A or COVID-19, 2020–2025*							
2020	Influenza A (3739)	45 (33–56)	1608 (43.0)	661 (17.7)	11 (0.3)	**Ref.**	**Ref.**
	COVID-19 (9371)	43 (30–57)	4396 (46.9)	1715 (18.3)	205 (2.2)	**6.92 (3.76–12.72)**	**6.11 (3.32–10.15)**
2021	Influenza A (458)	25 (21–40)	206 (45.0)	46 (10.0)	0 (0.0)	**Ref.**	**Ref.**
	COVID-19 (12 134)	42 (29–57)	5272 (43.4)	2513 (20.7)	213 (1.8)	**Too few flu cases/deaths to evaluate**	**Too few flu cases/deaths to evaluate**
2022	Influenza A (4841)	36 (25–54)	1832 (37.8)	791 (16.3)	19 (0.4)	**Ref.**	**Ref.**
	COVID-19 (25 265)	46 (32–61)	9676 (38.3)	6050 (23.9)	202 (0.8)	**2.01 (1.25–3.32)**	**1.35 (.83–2.20)**
2023	Influenza A (3770)	47 (35–60)	1493 (39.6)	896 (23.8)	13 (0.3)	**Ref.**	**Ref.**
	COVID-19 (11 652)	53 (37–68)	4257 (36.5)	3526 (30.3)	101 (0.9)	**2.51 (1.40–4.50)**	**1.49 (.82–2.73)**
2024	Influenza A (3988)	43 (29–59)	1615 (40.5)	833 (20.9)	17 (0.4)	**Ref.**	**Ref.**
	COVID-19 (12 046)	53 (37–68)	4374 (36.3)	3789 (31.5)	98 (0.8)	**1.90 (1.10–3.30)**	**1.03 (.59–1.79)**
2025	Influenza A (7875)	45 (31–61)	3147 (40.0)	1844 (23.4)	36 (0.5)	**Ref.**	**Ref.**
	COVID-19 (4550)	53 (36–69)	1596 (35.1)	1529 (33.6)	46 (1.0)	**2.20 (1.42–3.42)**	**1.23 (.76–2.00)**
*30-d mortality rates following a positive test for influenza A or COVID-19 detected by a respiratory viral battery, 2024–2025*							
2024–2025	Influenza A 2513	49 (32–64)	972 (38.7)	802 (31.9)	**29 (1.2)**	**Ref.**	**Ref.**
2024–2025	COVID-19 2627	56 (37–72)	984 (37.5)	1068 (40.7)	**45 (1.7)**	**1.52 (.95–2.43)**	**0.91 (.56–1.49)**

Abbreviations: CI, confidence interval; COVID-19, coronavirus disease 2019; IQR, interquartile range; OR, odds ratio.

Relative to influenza A, 30-day mortality following COVID-19 was higher in 2020 (adjusted OR [aOR] 6.11, 95% confidence interval [CI] 3.32–10.15), but the difference narrowed by 2025 (aOR 1.23, 95% CI .76–2.00; Table 1). There were too few influenza A observations in 2021 to support comparison for that year. The sensitivity analysis was limited to a single orderable test containing both COVID-19 and influenza A confirmed overall similar mortality rate between COVID-19 and influenza A in the 2024–2025 time period (Table 1). A summary of covariate effects used in the mortality adjustments between influenza and COVID-19 is provided in Supplementary Table 1: age, congestive heart failure, chronic kidney disease, and metastatic cancer were particularly strongly associated with mortality—justifying handling these as key confounders. As another post hoc sensitivity analysis, we also compared mortality rates following influenza A or COVID-19 in a propensity-matched cohort, which also found similar to marginally higher mortality rates following COVID-19 relative to influenza (Supplementary Table 2).

## DISCUSSION

In our single-center longitudinal survey of 30-day mortality following a positive COVID-19 test, we observed a decline in both crude and adjusted mortality rates from 2020 to 2025. Compared to influenza A, COVID-19 was associated with a higher initial mortality rate but has more recently stabilized, with adjusted odds for 30-day mortality ranging from 1.03 to 1.49 from 2022 onward. Similar decreases in mortality were reported as early as the first year of the pandemic, and our results are overall consistent with the recent Global Burden of Disease study in which COVID-19 began as a leading cause of mortality in 2020 but fell from the top 20 causes by 2025 [1, 11, 12]. Other studies noted similar changes in the population tested for COVID-19 over time but still observed decreasing mortality rates even after adjustment for potential confounding—consistent with our findings [13, 14].

Notably, the lesser mortality difference between COVID-19 and influenza A following multivariable adjustment supports our suspicion of confounding risk factors that differ between patients testing positive for each of these infections. Given evidence for confounding, it is likely that the adjusted mortality differences are closer to reality—for example, it seems likely that COVID-19 and influenza mortality became quite similar in 2024–2025 despite COVID-19's slightly higher crude mortality rate.

Our study has several strengths. We excluded tests performed for screening or administrative purposes to focus results on a clinically relevant population. Our dataset represents one of the largest sites for COVID-19 testing in our county and spans a 5-year period. Our ability to capture comorbidities and deaths was aided by the fact that most patients included in this cohort receive regular healthcare through our institution.

Our study carries several limitations. First, it is a retrospective laboratory-based survey. Although we attempted to adjust for changing demographics among individuals tested for COVID-19 over time, residual confounding cannot be excluded. For example, as testing became more widely available, it is possible that a greater proportion of milder ambulatory cases might shift estimated mortality rates lower. Additionally, given that vaccination, prior testing, and treatment could have been received in settings outside of our health care system, we were unable to account for the specific effects of vaccination, prior disease, or treatment. The observed decreases in mortality rates over time do temporally correspond with local availability of COVID-19 vaccines. Second, because our survey analyzed the tested population from 1 academic center, observed absolute mortality rates are unlikely to be generalizable to other populations. At least the relative trends in mortality are likely to be comparable to other institutions. Third, 30-day mortality queried through our electronic health record was all cause and not necessarily infection related. Additionally, while our electronic health record interfaces with death registries, some deaths occurring outside of our health system may still be missed. Fourth, comparison of COVID-19 to influenza A mortality rates may be confounded by pathogen-specific differences in mortality risk or decision to test.

In summary, while 30-day mortality following COVID-19 infection has trended downward since 2020, it remains numerically similar to slightly higher than influenza A through 2025 and shows no suggestion of any ongoing decrease. These data support the continued relevance of both COVID-19 and influenza A, particularly among those at higher risk of severe disease, and may aid in risk discussions for both patients and policymakers when considering vaccination or other infection prevention measures.

## Supplementary Material

ofag138_Supplementary_Data

## Supplementary Data

Supplementary materials are available at *Open Forum Infectious Diseases* online. Consisting of data provided by the authors to benefit the reader, the posted materials are not copyedited and are the sole responsibility of the authors, so questions or comments should be addressed to the corresponding author.
