# Supplementary material for: Trends in Coronavirus Disease 2019 Mortality Within a US Academic Health System, 2020–2025
Source: Open Forum Infect Dis. 2026 Mar 14;13(3):ofag138. doi: 10.1093/ofid/ofag138 (PMC13014464; doi:10.1093/ofid/ofag138)
Supplement: ofag138_Supplementary_Data [file ofag138_supplementary_data.docx]

**SUPPLEMENT**

**Table 1:** Comparative Modeling of 30-day Mortality Following a Positive Test for Influenza versus COVID-19

| Covariate | Univariate (unadjusted) Odds Ratios (95% CI) | Multivariate (adjusted) Odds Ratios (95% CI) |
| --- | --- | --- |
| Age, per decade | 2.34 (1.97-2.79) | 2.00 (1.60-2.50) |
| Male sex | 2.30 (1.46-3.64) | 1.85 (1.12-3.06) |
| Cancer | 4.03 (2.51-6.47) | 0.87 (0.48-1.60) |
| Complicated Diabetes | 2.54 (1.54-4.19) | 0.64 (0.35-1.18) |
| CHF | 9.03 (5.62-14.51) | 2.77 (1.48-5.18) |
| CKD | 9.04 (5.56-14.69) | 2.32 (1.23-4.35) |
| CTD | 2.10 (0.96-4.61) | 1.20 (0.51-2.87) |
| Dementia | 3.53 (1.60-7.77) | 1.04 (0.46-2.36) |
| HIV | 1.07 (0.15-7.92) | 1.28 (0.13-1.27) |
| Liver | 2.32 (1.26-4.27) | 1.30 (0.65-2.61) |
| Metastatic | 6.38 (3.36-12.10) | 3.44 (1.55-7.66) |
| Paresis | 3.96 (1.78-8.81) | 2.02 (0.86-4.76) |
| Pulmonary | 1.39 (0.87-2.19) | 0.76 (0.45-1.29) |
| Severe liver | 1.19 (0.16-8.77) | 0.75 (0.08-6.61) |
| SOT | 3.20 (0.97-10.52) | 1.38 (0.30-6.43) |
| HSCT | 4.75 (1.27-17.83) | 1.95 (0.19-19.93) |
| Infection |  |  |
| Influenza A | Reference | Reference |
| COVID-19 | 1.52 (0.95-2.43) | 0.91 (0.56-1.49) |

**Table 2:** 30-day Mortality Following a Positive Test for Influenza versus COVID-19 Within a Propensity-Matched Cohort of Subjects Undergoing Testing via Single Orderable Panel, 2024-2025

Briefly, a logistic regression model was constructed estimating the probability of testing positive for COVID-19 as opposed to influenza. This model was used to assign propensity scores to individual patients based on age, sex, cancer, complicated diabetes, congestive heart failure, chronic kidney disease, connective tissue disease, dementia, human immunodeficiency virus infection, liver disease, metastatic cancer, paresis, pulmonary disease, severe liver disease, solid organ transplant receipt, and hematopoietic stem cell transplant receipt. Subjects from the influenza and COVID-19 groups were matched 1:1 with a caliper of 0.1. Relative to the overall cohort, 4,614/5,140 (89.8%) of subjects were successfully matched.

| Matched Cohort | Mortality Rate (%) |
| --- | --- |
| Influenza | 29/2,278 (1.3) |
| COVID-19 | 32/2,275 (1.4) |
